# Supplementary material for: Growth, Metabolite, Antioxidative Capacity, Transcriptome, and the Metabolome Response to Dietary Choline Chloride in Pacific White Shrimp Litopenaeus vannamei
Source: Animals (Basel). 2020 Nov 30;10(12):2246. doi: 10.3390/ani10122246 (PMC7760581; doi:10.3390/ani10122246)
Supplement: Supplementary file 1 [file animals-10-02246-s001.pdf]

**Table S1.** The differential metabolites between shrimp given 0 and 6000mg/kg dietary choline.

| No. | Metabolite | M/Z | Formula | VIP (OPLS-DA) | FC (6000/0) | *P* value |
|---|---|---|---|---|---|---|
| 1 | 3,6-Ditigloyloxytropan-7-ol | 318.174 | C18H27NO5 | 3.5839 | 1.4836 | 0.04323 |
| 2 | L-2-Amino-3-methylenehexanoic acid | 350.2061 | C7H13NO2 | 2.6466 | 1.1623 | 0.002594 |
| 3 | PC(O-16:0/18:2(9Z,12Z)) | 788.5809 | C42H82NO7P | 2.596 | 1.1255 | 0.02227 |
| 4 | 13,14-Dihydro-15-keto-PGE2 | 351.2208 | C20H32O5 | 2.3443 | 1.0961 | 0.02059 |
| 5 | Mycosporine | 262.1275 | C11H19NO6 | 1.8346 | 1.0732 | 0.04879 |
| 6 | PE-NMe2(16:0/18:1(9Z)) | 790.5614 | C41H80NO8P | 1.956 | 1.0694 | 0.04182 |
| 7 | PE(15:0/20:1(11Z)) | 776.5448 | C40H78NO8P | 1.6237 | 1.0443 | 0.04722 |
| 8 | PE-NMe2(15:0/18:2(9Z,12Z)) | 774.5292 | C40H76NO8P | 1.6666 | 1.0399 | 0.01122 |
| 9 | PC(16:0/22:6(3Z,6Z,9Z,12Z,15Z,18))[U] | 806.5666 | C46H80NO8P | 1.0195 | 0.9849 | 0.03542 |
| 10 | PC(16:0/20:5(5Z,8Z,11Z,14Z,17Z)) | 780.5511 | C44H78NO8P | 1.0519 | 0.9841 | 0.03679 |
| 11 | PC(16:0/20:5(5E,8E,11E,14E,17E))[U] | 780.5488 | C44H78NO8P | 1.1621 | 0.9829 | 0.005741 |
| 12 | PC(18:1(11Z)/22:6(4Z,7Z,10Z,13Z,16Z,19Z)) | 832.5818 | C48H82NO8P | 1.1142 | 0.9818 | 0.03107 |
| 13 | Lamivudine sulfoxide | 266.0199 | C8H11N3O4S | 1.1888 | 0.9814 | 0.005128 |
| 14 | D-Pipecolic acid | 130.0859 | C6H11NO2 | 1.0474 | 0.9797 | 0.01148 |
| 15 | Bis(2-ethylhexyl) phthalate | 413.2648 | C24H38O4 | 1.1539 | 0.9783 | 0.04662 |
| 16 | PE-NMe2(18:0/20:5(5Z,8Z,11Z,14Z,17Z)) | 794.5664 | C45H80NO8P | 1.1501 | 0.9779 | 0.01903 |
| 17 | PC(18:3(6Z,9Z,12Z)/20:5(5Z,8Z,11Z,14Z,17Z)) | 802.5329 | C46H76NO8P | 1.2881 | 0.977 | 0.01091 |
| 18 | PC(18:0/20:5(5Z,8Z,11Z,14Z,17Z)) | 808.5818 | C46H82NO8P | 1.4866 | 0.9723 | 0.001586 |
| 19 | C17 Sphinganine | 288.2885 | C17H37NO2 | 1.2418 | 0.9709 | 0.01368 |
| 20 | L-2-Amino-5-hydroxypentanoic acid | 116.0705 | C5H11NO3 | 1.5021 | 0.965 | 0.02016 |
| 21 | LysoPE(18:0/0:0) | 480.3093 | C23H48NO7P | 1.7644 | 0.9621 | 0.003419 |
| 22 | Choline | 104.107 | C5H13NO | 1.8202 | 0.9586 | 0.0006582 |
| 23 | 2-Pyrrolidinone | 86.06028 | C4H7NO | 1.478 | 0.9575 | 0.01296 |
| 24 | 2-Mercapto-2-methyl-1-pentanol | 117.0738 | C6H14OS | 1.5397 | 0.9545 | 0.01937 |
| 25 | Acetyl-DL-Leucine | 172.0969 | C8H15NO3 | 1.5314 | 0.9534 | 0.01597 |
| 26 | Octadecanamide | 316.3198 | C18H37NO | 1.5609 | 0.9529 | 0.005178 |
| 27 | N-Stearoyl GABA | 352.3197 | C22H43NO3 | 1.7729 | 0.9497 | 0.0002882 |
| 28 | PE(18:0/0:0) | 482.3225 | C23H48NO7P | 1.778 | 0.949 | 0.004454 |
| 29 | L-Proline | 160.0347 | C5H9NO2 | 1.8356 | 0.9481 | 0.001248 |
| 30 | Porphobilinogen | 227.1017 | C10H14N2O4 | 1.6802 | 0.9479 | 0.006026 |
| 31 | Pipericine | 336.3246 | C22H41NO | 1.6236 | 0.9353 | 0.02678 |
| 32 | Ent-Prostaglandin F2alpha | 353.233 | C20H34O5 | 2.2297 | 0.925 | 0.02686 |
| 33 | 5-trans-PGE2 | 351.2173 | C20H32O5 | 2.2929 | 0.9193 | 0.04832 |
| 34 | 3-((3R,5S)-3-butyloctahydroindolizin-5-yl)propan-1-ol | 240.2314 | C15H29NO | 1.8873 | 0.9162 | 0.01805 |
| 35 | (4E,d14:1) sphingosine | 226.2157 | C14H29NO2 | 2.4117 | 0.9078 | 0.005845 |
| 36 | (2'E,4'Z,7'Z,8E)-Colnelenic acid | 334.2365 | C18H28O3 | 2.1824 | 0.905 | 0.003282 |
| 37 | 15-deoxy-delta-12,14-PGJ2 | 315.1961 | C20H28O3 | 2.4286 | 0.8867 | 0.04921 |
| 38 | C16 Sphingosine | 254.2468 | C16H33NO2 | 2.6533 | 0.8813 | 0.003957 |
| 39 | 12-epi Leukotriene B3 | 337.2382 | C20H34O4 | 3.4316 | 0.8509 | 0.003297 |
| 40 | 11-deoxy-PGE1 | 321.2412 | C20H34O4 | 2.9745 | 0.8453 | 0.005221 |
| 41 | LTB3 | 321.2412 | C20H34O4 | 3.1259 | 0.8364 | 0.006828 |
| 42 | 6-pentadecyl Salicylic Acid | 349.2725 | C22H36O3 | 3.8071 | 0.7637 | 0.0004888 |

Abbreviation: VIP, Variable Importance in the Projection; FC, fold change. FC >1, metabolites were upregulated in shrimp given 6000mg/kg dietary choline chloride.

**Table S2.** The differential metabolites between shrimp given 0 and 12000mg/kg dietary choline.

| No. | Metabolite | M/Z | Formula | VIP (OPLS-DA) | FC (12000/0) | *P* value |
|---|---|---|---|---|---|---|
| 1 | 3,6-Ditigloyloxytropan-7-ol | 318.174 | C18H27NO5 | 2.8316 | 1.3207 | 0.03123 |
| 2 | L-2-Amino-3-methylenehexanoic acid | 350.2061 | C7H13NO2 | 3.2235 | 1.2136 | 0.002773 |
| 3 | Angularine | 352.1776 | C18H25NO6 | 2.4069 | 1.1473 | 0.02984 |
| 4 | PAz-PC | 666.4323 | C33H64NO10P | 2.505 | 1.1395 | 0.006747 |
| 5 | Tetraphyllin B sulfate | 404.0076 | C12H17NO10S | 2.2492 | 1.1293 | 0.04526 |
| 6 | 13,14-Dihydro-15-keto-PGE2 | 351.2208 | C20H32O5 | 2.4558 | 1.1044 | 0.006433 |
| 7 | 4-Methylene-L-glutamine | 159.0758 | C6H10N2O3 | 1.9114 | 1.094 | 0.0176 |
| 8 | Capnine | 352.2502 | C17H37NO4S | 2.3706 | 1.0915 | 0.001307 |
| 9 | PE-NMe2(16:0/18:2(9Z,12Z)) | 788.5469 | C41H78NO8P | 1.909 | 1.071 | 0.02791 |
| 10 | (+)-cis-5,6-Dihydro-5-hydroxy-4-methoxy-6-(2-phenylethyl)-2H-pyran-2-one | 281.1373 | C14H16O4 | 1.8129 | 1.0693 | 0.02083 |
| 11 | PE-NMe2(16:0/18:1(9Z)) | 790.5614 | C41H80NO8P | 1.8526 | 1.0598 | 0.02368 |
| 12 | Alpha-Methylstyrene | 119.0852 | C9H10 | 1.6741 | 1.0574 | 0.0282 |
| 13 | N-Methyl-1H-indole-3-propanamide | 446.2523 | C12H14N2O | 1.7512 | 1.0536 | 0.01595 |
| 14 | Thromboxane B3 | 432.2367 | C20H32O6 | 1.7093 | 1.05 | 0.02494 |
| 15 | Nepsilon-Acetyl-L-lysine | 189.1227 | C8H16N2O3 | 1.6832 | 1.0447 | 0.000493 |
| 16 | PC(14:0/14:0) | 678.505 | C36H72NO8P | 1.4933 | 1.0442 | 0.02726 |
| 17 | 1H-Indole-3-acetamide | 157.0755 | C10H10N2O | 1.3287 | 1.0442 | 0.04048 |
| 18 | 5-Methyldeoxycytidine | 242.1125 | C10H15N3O4 | 1.4027 | 1.0405 | 0.03409 |
| 19 | Glycerophosphocholine | 280.091 | C8H20NO6P | 1.2392 | 1.0398 | 0.0405 |
| 20 | PE(15:0/20:1(11Z)) | 776.5448 | C40H78NO8P | 1.5581 | 1.0397 | 0.02704 |
| 21 | PC(15:0/18:2(9Z,12Z)) | 744.5513 | C41H78NO8P | 1.5203 | 1.0388 | 0.03536 |
| 22 | 2-octenoylglycine | 241.1537 | C10H17NO3 | 1.271 | 1.0286 | 0.02298 |
| 23 | Semilepidinoside B | 365.1349 | C17H22N2O7 | 1.1492 | 1.027 | 0.04733 |
| 24 | Cyclobrassinone | 213.0146 | C11H8N2O2S | 1.0146 | 1.0197 | 0.03098 |
| 25 | PC(14:0/18:1(11Z)) | 732.5516 | C40H78NO8P | 1.204 | 1.0196 | 0.01234 |
| 26 | Lamivudine sulfoxide | 266.0199 | C8H11N3O4S | 1.0537 | 0.984 | 0.003397 |
| 27 | PC(18:0/20:3(8Z,11Z,14Z)) | 834.5973 | C46H86NO8P | 1.3885 | 0.9776 | 0.000441 |
| 28 | N-Stearoyl GABA | 352.3197 | C22H43NO3 | 1.195 | 0.9772 | 0.002144 |
| 29 | C17 Sphinganine | 288.2885 | C17H37NO2 | 1.1208 | 0.9766 | 0.04475 |
| 30 | Tetraethylene glycol | 195.1221 | C8H18O5 | 1.2216 | 0.9736 | 0.01685 |
| 31 | Stearoylcarnitine | 464.3143 | C25H49NO4 | 1.4342 | 0.9627 | 0.03344 |
| 32 | Choline | 104.107 | C5H13NO | 1.7691 | 0.9579 | 0.006398 |
| 33 | PE(18:0/0:0) | 482.3225 | C23H48NO7P | 1.8534 | 0.9482 | 0.001486 |
| 34 | LysoPE(18:0/0:0) | 480.3093 | C23H48NO7P | 2.1029 | 0.9426 | 0.001735 |
| 35 | Fusarochromanone | 334.1747 | C15H20N2O4 | 1.628 | 0.9299 | 0.04096 |
| 36 | 3-((3R,5S)-3-butyloctahydroindolizin-5-yl)propan-1-ol | 240.2314 | C15H29NO | 1.7694 | 0.9247 | 0.01117 |
| 37 | 5-trans-PGE2 | 351.2173 | C20H32O5 | 2.8503 | 0.8946 | 0.005207 |
| 38 | (4E,d14:1) sphingosine | 226.2157 | C14H29NO2 | 2.6886 | 0.8914 | 0.001027 |
| 39 | Ent-Prostaglandin F2alpha | 353.233 | C20H34O5 | 2.7004 | 0.8866 | 0.01846 |
| 40 | C16 Sphingosine | 254.2468 | C16H33NO2 | 2.7366 | 0.868 | 0.002026 |
| 41 | 15-deoxy-delta-12,14-PGJ2 | 315.1961 | C20H28O3 | 2.9458 | 0.8552 | 0.007009 |

| | | | | | | |
|---|---|---|---|---|---|---|
| 42 | 11-deoxy-PGE1 | 321.2412 | C20H34O4 | 2.6204 | 0.8549 | 0.03317 |
| 43 | 12-epi Leukotriene B3 | 337.2382 | C20H34O4 | 3.3125 | 0.8509 | 0.002524 |
| 44 | LTB3 | 321.2412 | C20H34O4 | 3.3008 | 0.8305 | 0.002658 |
| 45 | 6-pentadecyl Salicylic Acid | 349.2725 | C22H36O3 | 4.0106 | 0.7564 | 8.72E-05 |

Abbreviation: VIP, Variable Importance in the Projection; FC, fold change. FC >1, metabolites were upregulated in shrimp given 12000mg/kg dietary choline chloride.

**Table S3.** The differential metabolites between shrimp given 6000 and 12000mg/kg dietary choline.

| No. | Metabolite | M/Z | Formula | VIP (OPLS-DA) | FC (12000/6000) | *P* value |
|---|---|---|---|---|---|---|
| 1 | 2-S-cysteinyl-DOPA | 281.057938 | C12H16N2O6S | 2.3258 | 1.1367 | 0.04626 |
| 2 | 4-Methylene-L-glutamine | 159.0758403 | C6H10N2O3 | 2.3513 | 1.1286 | 0.006801 |
| 3 | N-(gamma-Glutamyl)ethanolamine | 155.0808709 | C7H14N2O4 | 2.171 | 1.1062 | 0.015 |
| 4 | Furaneol 4-glucoside | 255.0884998 | C12H18O8 | 1.8562 | 1.0775 | 0.02426 |
| 5 | N-Methyl-1H-indole-3-propanamide | 446.2522699 | C12H14N2O | 1.8358 | 1.0573 | 0.02835 |
| 6 | Thromboxane B3 | 432.236738 | C20H32O6 | 1.7391 | 1.051 | 0.04967 |
| 7 | PC(15:0/20:3(5Z,8Z,11Z)) | 770.566584 | C43H80NO8P | 1.5313 | 1.0449 | 0.04953 |
| 8 | PC(14:0/14:0) | 678.5049518 | C36H72NO8P | 1.4946 | 1.0433 | 0.04528 |
| 9 | N-(5-Methyl-3-oxohexyl)alanine | 202.1430469 | C10H19NO3 | 1.2676 | 1.0428 | 0.04504 |
| 10 | (S)-Succinyldihydrolipoamide | 352.0618263 | C12H21NO4S2 | 1.2132 | 1.0381 | 0.04474 |
| 11 | N-ACETYLPROLINE | 158.0806144 | C7H11NO3 | 1.0908 | 1.0373 | 0.04508 |
| 12 | (R)-(+)-2-Pyrrolidone-5-carboxylic acid | 130.0495223 | C5H7NO3 | 1.434 | 1.0357 | 0.01041 |
| 13 | PE-NMe2(18:0/20:5(5Z,8Z,11Z,14Z,17Z)) | 794.5663656 | C45H80NO8P | 1.3973 | 1.0349 | 0.03429 |
| 14 | L-Proline | 160.034699 | C5H9NO2 | 1.2391 | 1.0334 | 0.02064 |
| 15 | N-Stearoyl GABA | 352.319661 | C22H43NO3 | 1.1179 | 1.029 | 0.02509 |
| 16 | Alpha-(Methylenecyclopropyl)glycine | 128.0702467 | C6H9NO2 | 1.0934 | 1.0284 | 0.03409 |
| 17 | 2-octenoylglycine | 241.1537007 | C10H17NO3 | 1.1985 | 1.0255 | 0.03098 |
| 18 | L-4-Hydroxyglutamate semialdehyde | 128.0339403 | C5H9NO4 | 1.3387 | 1.0247 | 0.03434 |
| 19 | PE(18:0/22:6(4Z,7Z,10Z,13Z,16Z,19Z)) | 805.5516927 | C45H78NO8P | 1.176 | 1.0245 | 0.04895 |
| 20 | PC(14:0/18:1(11Z)) | 732.5515592 | C40H78NO8P | 1.296 | 1.0243 | 0.016 |
| 21 | PC(18:1(11Z)/18:2(9Z,12Z)) | 784.5818936 | C44H82NO8P | 1.2547 | 1.0218 | 0.02587 |
| 22 | PC(15:0/20:2(11Z,14Z)) | 772.5824567 | C43H82NO8P | 1.0812 | 1.0196 | 0.02817 |
| 23 | N-[2-(3,4-dihydroxyphenyl)ethyl]-3-(4-hydroxy-3-methoxyphenyl)propanimidic acid | 330.1378104 | C18H21NO5 | 2.6935 | 0.8814 | 0.02959 |

Abbreviation: VIP, Variable Importance in the Projection; FC, fold change. FC >1, metabolites were upregulated in shrimp given 12000mg/kg dietary choline chloride.

**Table S4.** The differential expression genes (DEGs) between shrimp given 0 and 6000mg/kg dietary choline.

| No. | Gene name | Gene description | $Log_2FC$ (6000/0) | *P* adjust | Regulate |
|---|---|---|---|---|---|
| 1 | LOC113810108 | anti-lipopolysaccharide factor-like | 7.844017 | 0.000111 | up |
| 2 | LOC113825867 | myosin heavy chain, cardiac muscle isoform-like | 5.787739 | 0.000863 | up |
| 3 | LOC113804544 | balbiani ring protein 3-like | 5.458167 | 0.04514 | up |
| 4 | LOC113815764 | molt-inhibiting hormone-like, transcript variant X1 | 5.096405 | 0.035458 | up |
| 5 | LOC113800626 | peritrophin-1-like | 4.807213 | 0.012688 | up |
| 6 | LOC113825869 | trichohyalin-like | 4.693693 | 4.12E-06 | up |
| 7 | LOC113825865 | GRIP and coiled-coil domain-containing protein 2-like | 4.170998 | 0.000183 | up |
| 8 | LOC113807950 | transferrin-like | 4.168086 | 3.19E-05 | up |
| 9 | LOC113820924 | ETS homologous factor-like | 4.051601 | 0.004139 | up |
| 10 | LOC113825866 | myosin-9-like | 3.656412 | 0.000232 | up |
| 11 | LOC113809104 | myosin-3-like, transcript variant X1 | 3.532677 | 0.021486 | up |
| 12 | LOC113803667 | leukocyte elastase inhibitor-like | 3.354274 | 0.044791 | up |
| 13 | LOC113800111 | peritrophin-1-like | 3.040403 | 0.011662 | up |
| 14 | LOC113825871 | myosin heavy chain, cardiac muscle isoform-like, transcript variant X1 | 3.016526 | 0.007911 | up |
| 15 | LOC113816489 | sodium-dependent nutrient amino acid transporter 1-like | 2.437762 | 0.018893 | up |
| 16 | LOC113813386 | differentially expressed in FDCP 8-like, transcript variant X1 | 2.12623 | 0.03013 | up |
| 17 | LOC113807093 | calponin homology domain-containing protein DDB_G0272472-like | 1.425283 | 0.04982 | up |
| 18 | LOC113819483 | diacylglycerol kinase epsilon-like, transcript variant X3 | -1.25709 | 0.026903 | down |
| 19 | LOC113810053 | zonadhesin-like | -1.52305 | 0.023393 | down |
| 20 | LOC113826143 | transient receptor potential channel pyrexia-like | -1.58665 | 0.033123 | down |
| 21 | LOC113827567 | nidogen-2-like | -1.70877 | 0.007347 | down |
| 22 | LOC113826144 | titin-like | -1.92808 | 0.004139 | down |
| 23 | LOC113813703 | microfibril-associated glycoprotein 4-like | -2.29295 | 0.001389 | down |
| 24 | LOC113805827 | carboxypeptidase A1-like | -2.36227 | 0.029292 | down |
| 25 | LOC113828069 | decorin-like | -2.4468 | 0.002579 | down |
| 26 | LOC113813817 | apolipoprotein D-like | -2.89322 | 1.54E-10 | down |
| 27 | LOC113808791 | oplophorus-luciferin 2-monooxygenase non-catalytic subunit-like | -2.92495 | 0.035884 | down |
| 28 | LOC113819957 | macrophage mannose receptor 1-like | -2.98044 | 0.024847 | down |
| 29 | LOC113809173 | perlucin-like protein | -3.2117 | 0.02006 | down |
| 30 | LOC113809991 | L-lactate dehydrogenase-like, transcript variant X2 | -3.41541 | 0.02006 | down |
| 31 | LOC113825544 | alpha-L-fucosidase-like, transcript variant X1 | -4.44381 | 0.004139 | down |
| 32 | LOC113813877 | heme-binding protein 2-like | -4.84094 | 0.039969 | down |
| 33 | LOC113813873 | heme-binding protein 2-like | -4.85959 | 0.000119 | down |
| 34 | LOC113810344 | heme-binding protein 2-like | -4.89099 | 0.034227 | down |
| 35 | LOC113827864 | legumain-like | -5.37446 | 0.02006 | down |
| 36 | LOC113825084 | nucleolar and coiled-body phosphoprotein 1-like | -5.40186 | 0.002596 | down |
| 37 | LOC113813869 | heme-binding protein 2-like | -5.49063 | 0.017872 | down |
| 38 | LOC113822187 | solute carrier family 15 member 2-like, transcript | -5.67644 | 0.000734 | down |

| | | | | | |
|---|---|---|---|---|---|
| | | variant X1 | | | |
| 39 | LOC113813588 | sodium-dependent nutrient amino acid transporter 1-like | -5.72589 | 0.004139 | down |
| 40 | LOC113801169 | hormone receptor 4-like, transcript variant X1 | -5.92713 | 0.000292 | down |
| 41 | LOC113807681 | b(0,+)-type amino acid transporter 1-like | -6.09243 | 0.001117 | down |
| 42 | LOC113820757 | perlucin-like protein | -6.10047 | 0.002238 | down |
| 43 | LOC113830327 | methylglutaconyl-CoA hydratase, mitochondrial-like | -6.24728 | 0.04287 | down |
| 44 | LOC113815264 | bile salt-activated lipase-like | -6.50885 | 0.008475 | down |
| 45 | LOC113810374 | heme-binding protein 2-like | -7.18469 | 1.22E-06 | down |
| 46 | LOC113810377 | heme-binding protein 2-like | -7.25731 | 0.022913 | down |
| 47 | LOC113813881 | heme-binding protein 2-like | -7.70394 | 0.004163 | down |
| 48 | LOC113813875 | heme-binding protein 2-like | -7.85354 | 0.007157 | down |
| 49 | LOC113830401 | organic cation transporter protein-like | -7.95365 | 0.004662 | down |
| 50 | LOC113813867 | heme-binding protein 2-like | -8.41596 | 0.000465 | down |
| 51 | LOC113813866 | heme-binding protein 2-like, transcript variant X1 | -8.44133 | 0.000388 | down |
| 52 | LOC113819956 | macrophage mannose receptor 1-like, transcript variant X1 | -9.0643 | 1.24E-07 | down |
| 53 | LOC113813597 | sodium-dependent nutrient amino acid transporter 1-like | -9.13228 | 1.11E-06 | down |
| 54 | LOC113823268 | facilitated trehalose transporter Tret1-2 homolog, transcript variant X1 | -10.4695 | 0.026903 | down |

Abbreviation: FC, fold change. $Log_2FC > 0$, DEGs were upregulated in shrimp given 6000mg/kg dietary choline chloride.

**Table S5.** The differential expression genes (DEGs) between shrimp given 0 and 12000mg/kg dietary choline.

| No. | Gene name | Gene description | $Log_2FC$ (12000/0) | *P* adjust | Regulate |
|---|---|---|---|---|---|
| 1 | LOC113827106 | 28S ribosomal protein S22, mitochondrial-like | 5.95414 | 4.21E-05 | up |
| 2 | LOC113821248 | indole-3-acetaldehyde oxidase-like | 4.324374 | 0.01008 | up |
| 3 | LOC113804075 | calcium-activated chloride channel regulator 1-like | 3.1413 | 0.041365 | up |
| 4 | LOC113811900 | GATA zinc finger domain-containing protein 10-like | 2.929035 | 0.011643 | up |
| 5 | LOC113824772 | chromatin-remodeling ATPase INO80-like | 2.269296 | 0.04208 | up |
| 6 | LOC113812427 | 3-oxoacyl-[acyl-carrier-protein] reductase FabG-like | 2.14331 | 0.032453 | up |
| 7 | LOC113824965 | period circadian protein-like | 1.800082 | 0.00389 | up |
| 8 | LOC113828937 | period circadian protein-like, transcript variant X1 | 1.703999 | 0.014564 | up |
| 9 | LOC113827646 | serine/threonine-protein phosphatase 6 regulatory ankyrin repeat subunit C-like | 1.651592 | 0.041365 | up |
| 10 | LOC113804391 | nuclear factor interleukin-3-regulated protein-like | 1.220087 | 0.015099 | up |
| 11 | LOC113818954 | N-acetylneuraminate lyase-like | -2.03882 | 0.035011 | down |
| 12 | LOC113813817 | apolipoprotein D-like | -2.13075 | 0.01008 | down |
| 13 | LOC113815249 | metallothionein-1-like | -2.16507 | 0.01008 | down |
| 14 | LOC113804871 | zinc finger protein 665-like | -2.45801 | 0.004784 | down |
| 15 | LOC113815366 | carotenoid isomerooxygenase-like | -3.00587 | 0.001461 | down |
| 16 | LOC113828559 | branchpoint-bridging protein-like, transcript variant X1 | -3.21203 | 0.000813 | down |
| 17 | LOC113813873 | heme-binding protein 2-like | -3.22311 | 0.000142 | down |
| 18 | LOC113828746 | TRIO and F-actin-binding protein-like | -3.29939 | 0.000647 | down |
| 19 | LOC113815038 | sphingomyelin phosphodiesterase-like | -3.30807 | 0.032453 | down |
| 20 | LOC113827839 | zyxin-like | -3.5391 | 1.09E-06 | down |
| 21 | LOC113828299 | cyclin-dependent kinase inhibitor 1C-like | -3.64207 | 4.2E-05 | down |
| 22 | LOC113824723 | crustacyanin-A2 subunit-like | -3.66914 | 0.001193 | down |
| 23 | LOC113828474 | formin-2-like | -3.70746 | 0.01008 | down |
| 24 | LOC113815367 | beta,beta-carotene 15,15'-dioxygenase-like | -3.83031 | 0.000853 | down |
| 25 | LOC113824728 | crustacyanin-A1 subunit-like | -3.98163 | 0.000813 | down |
| 26 | LOC113824726 | crustacyanin-A2 subunit-like | -4.15613 | 8.97E-05 | down |
| 27 | LOC113824735 | crustacyanin-A2 subunit-like | -4.22246 | 0.00389 | down |
| 28 | LOC113824722 | crustacyanin-A2 subunit-like | -4.4782 | 2.12E-09 | down |
| 29 | LOC113824721 | crustacyanin-C1 subunit-like | -4.56512 | 8.63E-05 | down |
| 30 | LOC113800930 | low-density lipoprotein receptor 1-like | -4.60708 | 0.041365 | down |
| 31 | LOC113806288 | leukocyte elastase inhibitor-like | -5.19245 | 3.19E-05 | down |
| 32 | LOC113824731 | crustacyanin-C1 subunit-like | -5.32356 | 0.004105 | down |
| 33 | LOC113820707 | protein patched homolog 2-like | -5.4304 | 0.008441 | down |
| 34 | LOC113824736 | crustacyanin-A2 subunit-like | -5.84059 | 0.000853 | down |
| 35 | LOC113823683 | probable cytosolic oligopeptidase A | -7.05389 | 0.000911 | down |
| 36 | LOC113800662 | glutathione S-transferase 1-like | -9.40786 | 0.000894 | down |

Abbreviation: FC, fold change. $Log_2FC$ >0, DEGs were upregulated in shrimp given 12000mg/kg dietary choline chloride.

**Table S6.** The differential expression genes (DEGs) between shrimp given 6000 and 12000mg/kg dietary choline.

| No. | Gene name | Gene description | $Log_2FC$ (12000/6000) | *P* adjust | Regulate |
|---|---|---|---|---|---|
| 1 | LOC113805072 | spore coat protein SP65-like | 13.49988 | 4.2E-13 | up |
| 2 | LOC113813597 | sodium-dependent nutrient amino acid transporter 1-like | 10.597c94 | 0.000395 | up |
| 3 | LOC113819956 | macrophage mannose receptor 1-like, transcript variant X1 | 9.987259 | 3.79E-07 | up |
| 4 | LOC113830401 | organic cation transporter protein-like | 9.950206 | 0.022393 | up |
| 5 | LOC113822187 | solute carrier family 15 member 2-like, transcript variant X1 | 8.691168 | 1.9E-06 | up |
| 6 | LOC113807681 | b(0,+)-type amino acid transporter 1-like | 8.012962 | 0.000454 | up |
| 7 | LOC113830327 | methylglutaconyl-CoA hydratase, mitochondrial-like | 7.175249 | 4.48E-09 | up |
| 8 | LOC113801169 | hormone receptor 4-like, transcript variant X1 | 6.556356 | 5.37E-05 | up |
| 9 | LOC113814499 | probable nuclear hormone receptor HR3, transcript variant X1 | 6.552909 | 9.25E-05 | up |
| 10 | LOC113806519 | mucin-5AC-like | 5.91834 | 0.001013 | up |
| 11 | LOC113826635 | ADP-ribosylation factor-like protein 5B | 5.903299 | 0.032149 | up |
| 12 | LOC113823254 | dnaJ homolog subfamily C member 9-like | 5.858019 | 0.045734 | up |
| 13 | LOC113818833 | excitatory amino acid transporter 1-like | 4.455748 | 0.00944 | up |
| 14 | LOC113825544 | alpha-L-fucosidase-like, transcript variant X1 | 4.275865 | 0.001377 | up |
| 15 | LOC113809991 | L-lactate dehydrogenase-like, transcript variant X2 | 3.643715 | 0.007221 | up |
| 16 | LOC113827830 | suppressor of cytokine signaling 2-like | 3.507174 | 0.043773 | up |
| 17 | LOC113823026 | alpha-(1,6)-fucosyltransferase-like | 3.487596 | 0.022199 | up |
| 18 | LOC113808791 | oplophorus-luciferin 2-monooxygenase non-catalytic subunit-like | 3.48378 | 0.038799 | up |
| 19 | LOC113815331 | excitatory amino acid transporter 3-like, transcript variant X1 | 3.460427 | 0.032912 | up |
| 20 | LOC113825741 | mannosylglucosyl-3-phosphoglycerate phosphatase-like | 3.382708 | 0.032713 | up |
| 21 | LOC113811889 | leucine-rich repeat neuronal protein 3-like, transcript variant X2 | 3.297297 | 0.032912 | up |
| 22 | LOC113808891 | phosphatidylserine decarboxylase proenzyme, mitochondrial-like, transcript variant X1 | 3.178162 | 0.034475 | up |
| 23 | LOC113825944 | uracil phosphoribosyltransferase homolog, transcript variant X1 | 3.171833 | 0.009126 | up |
| 24 | LOC113827042 | testicular acid phosphatase homolog | 3.102037 | 0.041045 | up |
| 25 | LOC113823490 | xaa-Pro aminopeptidase 1-like | 2.848834 | 0.029871 | up |
| 26 | LOC113802825 | glutamine--fructose-6-phosphate aminotransferase [isomerizing] 2-like, transcript variant X1 | 2.793581 | 0.04657 | up |
| 27 | LOC113811776 | baculoviral IAP repeat-containing protein 8-like | 2.744965 | 0.049244 | up |
| 28 | LOC113819860 | sodium/glucose cotransporter 5-like | 2.723348 | 0.032622 | up |
| 29 | LOC113806747 | aquaporin AQPAe.a-like | 2.669321 | 0.018569 | up |
| 30 | LOC113823293 | caspase-1-like, transcript variant X1 | 2.587017 | 0.008012 | up |
| 31 | LOC113818103 | failed axon connections homolog | 2.5865 | 0.038799 | up |
| 32 | LOC113805827 | carboxypeptidase A1-like | 2.375335 | 0.000206 | up |
| 33 | LOC113818038 | arylsulfatase B-like | 2.317479 | 0.003841 | up |
| 34 | LOC113821424 | protein extra-macrochaetae-like | 2.302437 | 0.003841 | up |
| 35 | LOC113805277 | ecdysone receptor-like, transcript variant X1 | 2.239864 | 0.003195 | up |
| 36 | LOC113814440 | ornithine decarboxylase antizyme 1-like | 2.018943 | 0.000693 | up |

| | | | | | |
|---|---|---|---|---|---|
| 37 | LOC113806944 | serine/arginine repetitive matrix protein 1-like | 1.971655 | 0.022199 | up |
| 38 | LOC113818477 | nose resistant to fluoxetine protein 6-like | 1.308009 | 0.041879 | up |
| 39 | LOC113812170 | ketimine reductase mu-crystallin-like, transcript variant X4 | 1.269424 | 0.003657 | up |
| 40 | LOC113804459 | carboxypeptidase Q-like | 1.207132 | 0.003841 | up |
| 41 | LOC113813092 | von Willebrand factor A domain-containing protein 7-like | 1.155607 | 0.020402 | up |
| 42 | LOC113828197 | EF-hand calcium-binding domain-containing protein 7-like | 1.123342 | 0.029765 | up |
| 43 | LOC113825899 | transient receptor potential cation channel subfamily A member 1 homolog, transcript variant X3 | 1.108051 | 0.04284 | up |
| 44 | LOC113812978 | macrophage mannose receptor 1-like | -1.33524 | 0.00062 | down |
| 45 | LOC113809352 | venom toxin OcyC11-like | -1.43839 | 0.002811 | down |
| 46 | LOC113815249 | metallothionein-1-like | -1.44626 | 0.048925 | down |
| 47 | LOC113809900 | sialin-like, transcript variant X1 | -1.63684 | 0.04806 | down |
| 48 | LOC113817695 | balbiani ring protein 3-like | -1.76205 | 0.003841 | down |
| 49 | LOC113827090 | phenoloxidase 3-like | -1.97695 | 0.019026 | down |
| 50 | LOC113826663 | golgin subfamily A member 6-like protein 22, transcript variant X1 | -2.306 | 0.017903 | down |
| 51 | LOC113828299 | cyclin-dependent kinase inhibitor 1C-like | -2.42734 | 0.001732 | down |
| 52 | LOC113818328 | multifunctional protein ADE2-like | -2.51618 | 0.031564 | down |
| 53 | LOC113813947 | facilitated trehalose transporter Tret1-like | -2.52103 | 0.003934 | down |
| 54 | LOC113826593 | alpha-1-inhibitor 3-like | -2.62332 | 0.034892 | down |
| 55 | LOC113800363 | anti-lipopolysaccharide factor-like | -2.68183 | 0.031564 | down |
| 56 | LOC113802295 | lysozyme C-like | -2.69754 | 0.047908 | down |
| 57 | LOC113825609 | sialin-like, transcript variant X1 | -2.79306 | 0.00062 | down |
| 58 | LOC113823795 | trypsin-1-like | -2.7932 | 0.006343 | down |
| 59 | LOC113810916 | delta(7)-sterol 5(6)-desaturase-like | -2.88896 | 0.017103 | down |
| 60 | LOC113809687 | alpha-1-inhibitor 3-like | -2.92052 | 0.000315 | down |
| 61 | LOC113815775 | WAP four-disulfide core domain protein 5-like | -2.93304 | 0.012603 | down |
| 62 | LOC113812834 | transforming growth factor-beta-induced protein ig-h3-like | -2.99105 | 0.015534 | down |
| 63 | LOC113802496 | phenoloxidase-activating factor 2-like | -3.05477 | 0.012063 | down |
| 64 | LOC113811681 | myc-associated zinc finger protein-like | -3.22772 | 0.028102 | down |
| 65 | LOC113828559 | branchpoint-bridging protein-like, transcript variant X1 | -3.31883 | 6.54E-05 | down |
| 66 | LOC113824726 | crustacyanin-A2 subunit-like | -3.31696 | 0.034475 | down |
| 67 | LOC113827839 | zyxin-like | -3.35266 | 1.14E-09 | down |
| 68 | LOC113826214 | phenoloxidase-activating factor 3-like | -3.41042 | 0.017903 | down |
| 69 | LOC113828746 | TRIO and F-actin-binding protein-like | -3.47993 | 0.000332 | down |
| 70 | LOC113804438 | type-2 ice-structuring protein-like | -3.46708 | 0.027005 | down |
| 71 | LOC113814992 | translation initiation factor IF-2-like | -3.53181 | 0.043773 | down |
| 72 | LOC113815367 | beta,beta-carotene 15,15'-dioxygenase-like | -3.82866 | 0.031418 | down |
| 73 | LOC113824723 | crustacyanin-A2 subunit-like | -3.88406 | 0.000421 | down |
| 74 | LOC113827875 | alpha-1-inhibitor 3-like | -4.02009 | 0.002744 | down |
| 75 | LOC113826189 | O-acyltransferase like protein-like | -4.01851 | 0.019026 | down |
| 76 | LOC113824728 | crustacyanin-A1 subunit-like | -4.04042 | 0.006632 | down |
| 77 | LOC113828474 | formin-2-like | -4.14341 | 1.39E-13 | down |
| 78 | LOC113803689 | uncharacterized protein PF11_0207-like, transcript variant X1 | -1.69567 | 0.000299 | down |
| 79 | LOC113815366 | carotenoid isomerooxygenase-like | -4.15222 | 0.000118 | down |

| | | | | | |
|---|---|---|---|---|---|
| 80 | LOC113818743 | protein white-like | -4.16391 | 0.001112 | down |
| 81 | LOC113824721 | crustacyanin-C1 subunit-like | -4.27014 | 0.000192 | down |
| 82 | LOC113805652 | CD209 antigen-like protein E | -4.37184 | 0.031564 | down |
| 83 | LOC113828724 | microtubule-associated protein futsch-like | -4.53329 | 0.000996 | down |
| 84 | LOC113823186 | sulfotransferase 1C4-like, transcript variant X1 | -4.78028 | 0.034475 | down |
| 85 | LOC113824731 | crustacyanin-C1 subunit-like | -4.84418 | 0.023571 | down |
| 86 | LOC113824736 | crustacyanin-A2 subunit-like | -5.16275 | 0.000693 | down |
| 87 | LOC113827177 | alcohol dehydrogenase [NADP(+)]-like | -5.29038 | 0.010814 | down |
| 88 | LOC113807647 | alpha-mannosidase 2C1-like | -5.38115 | 0.036814 | down |
| 89 | LOC113806288 | leukocyte elastase inhibitor-like | -5.83852 | 4.77E-10 | down |
| 90 | LOC113800744 | nucleoside diphosphate-linked moiety X motif 8-like | -6.36704 | 0.008748 | down |
| 91 | LOC113823683 | probable cytosolic oligopeptidase A | -8.28377 | 0.003483 | down |

Abbreviation: FC, fold change. $Log_2FC > 0$, DEGs were upregulated in shrimp given 12000mg/kg dietary choline chloride.

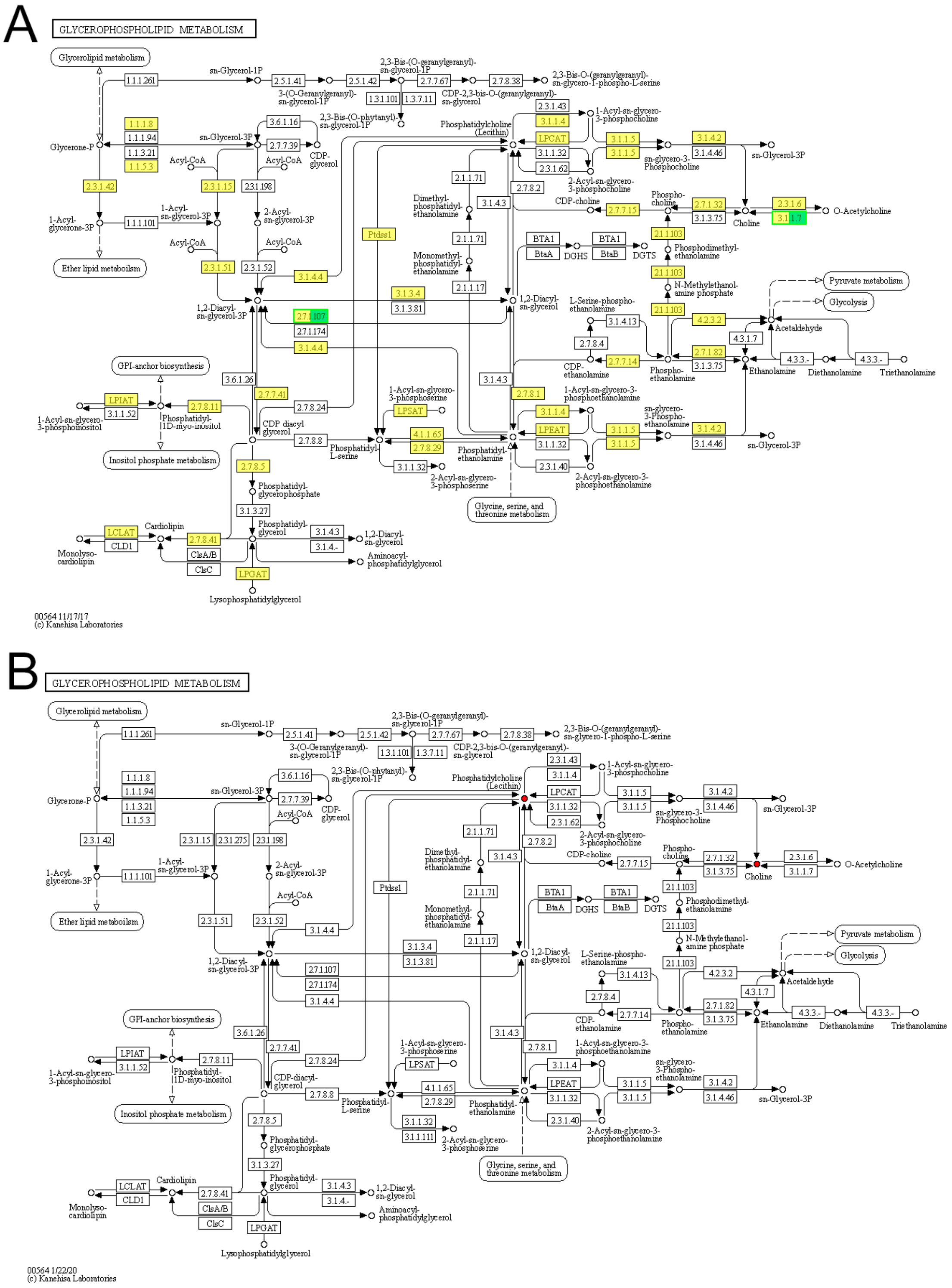


**Figure S1.** The KEGG pathway enrichment analysis for glycerophospholipid lipid pathway between shrimp fed 0 and 6000 mg/kg dietary choline (A, transcriptome; B, metabolome). Green squares represent downregulated genes in shrimp fed 6000 mg/kg choline. Red circle represent downregulated metabolites in shrimp fed 6000 mg/kg choline.
